# Enhanced Grüneisen Parameter in Supercooled Water

**DOI:** 10.1038/s41598-019-48353-4

**Published:** 2019-08-19

**Authors:** Gabriel O. Gomes, H. Eugene Stanley, Mariano de Souza

**Affiliations:** 1 University of São Paulo, Department of Astronomy, São Paulo, 05508-090 Brazil; 2 Boston University, Department of Physics, Boston, 02215 USA; 3 São Paulo State University, IGCE - Department of Physics, Rio Claro, SP 13506-900 Brazil

**Keywords:** Physics, Phase transitions and critical phenomena, Phase transitions and critical phenomena

## Abstract

We use the recently-proposed *compressible cell* Ising-like model to estimate the ratio between thermal expansivity and specific heat (the Grüneisen parameter Γ_*s*_) in supercooled water. Near the critical pressure and temperature, Γ_*s*_ becomes significantly sensitive to thermal fluctuations of the order-parameter, a characteristic behavior of pressure-induced critical points. Such enhancement of Γ_*s*_ indicates that two energy scales are governing the system, namely the coexistence of high- and low-density liquids, which become indistinguishable at the critical point in the supercooled phase. The temperature dependence of the compressibility, sound velocity and pseudo-Grüneisen parameter Γ_*w*_ are also reported. Our findings support the proposed liquid-liquid critical point in supercooled water in the No-Man’s Land regime, and indicates possible applications of this model to other systems. In particular, an application of the model to the qualitative behavior of the Ising-like nematic phase in Fe-based superconductors is also presented.

## Introduction

**Figure 1:**
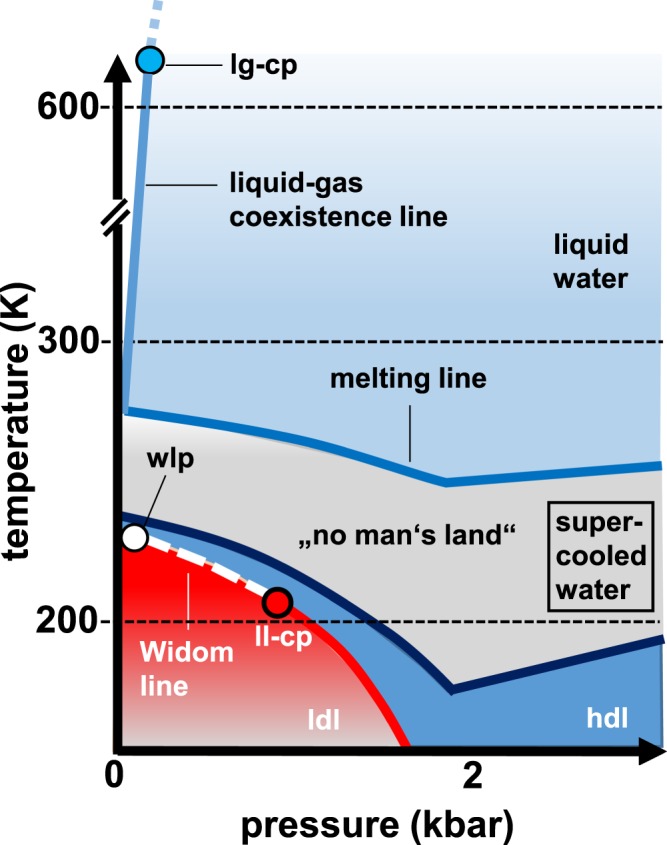
Temperature *versus* pressure phase diagram of water, lg-cp refers to liquid-gas critical point, wlp is the Widom line point and ll-cp indicates the liquid-liquid critical point, which is the focus of the present work. We have used in our analysis ${T}_{c}\simeq 180\,{\rm{K}}$, see main text. Picture after^2,6^.

Because it is biologically fundamental to the maintenance of all life, liquid water is one of the most important substances on the planet. Water exhibits a number of anomalous physical properties (see Fig. 1, refs^1,2^ and references cited therein), and over the last 25 years, much attention has been paid to the study of water on its so-called supercooled phase. The initial work on supercooled water in 1992 used molecular dynamics simulations^3^. A subsequent research has explored the No-Man’s Land region in the phase diagram (see Fig. 1 and ref.^2^). This topic has generated much debate (cf. refs^2,4–7^ and references therein).

One scenario describing supercooled water assumes the existence of two liquid phases at low-*T*, being each phase associated with either a high- or low-density^6^. Recently fs x-ray scattering was used on water droplets to determine the maximum isothermal compressibility, the correlation length, and the structures of water and heavy water. Experimental evidence of a second-order critical end-point in the Widom line was found^7^, but no clear-cut divergence in the quantities was observed. Here we study the liquid-liquid critical point for supercooled water by analysing the behavior of the Grüneisen parameter (${{\rm{\Gamma }}}_{s}$), see Methods. Such approach has already been successfully applied to other systems^8–11^. In the case of supercooled water, we find evidence supporting a liquid-liquid critical point. We use a recently-proposed *compressible cell* Ising-like model^12–14^ to obtain ${{\rm{\Gamma }}}_{s}$. Essentially, the model proposed in ref.^12^ assumes the coexistence of two possible volume values for each cell on the lattice, represented by ${v}_{-}={v}_{0}$ and ${v}_{+}={v}_{0}+\delta v$. These volumes are responsible for the change between high- and low-density liquids in the system. The two free volumes, i.e., the volume where a particle inside the cell can move, for each cell are $0 < {\dot{v}}_{+} < {v}_{+}$ and $0 < {\dot{v}}_{-} < {v}_{-}$ and their ratio is $\lambda ={\dot{v}}_{+}/{\dot{v}}_{-}$, see Methods.

It is worth mentioning that a structurally similar model was originally proposed in ref.^15^ and employed in the study of a large number of fluids, cf. ref.^16^. Taking such studies into account, we emphasize that the originality of the present work lies not only on the choice of the model for the analysis of supercooled water, but also on its application in the analysis of ${{\rm{\Gamma }}}_{s}$ to a regime where experimental results are lacking as a consequence of the rapid crystallization of water under such conditions^17^. Our analysis of ${{\rm{\Gamma }}}_{s}$ is complemented by the discussion of the pseudo-Grüneisen parameter (${{\rm{\Gamma }}}_{w}$)^18^, see Methods.

## Results and Discussion

**Figure 2:**
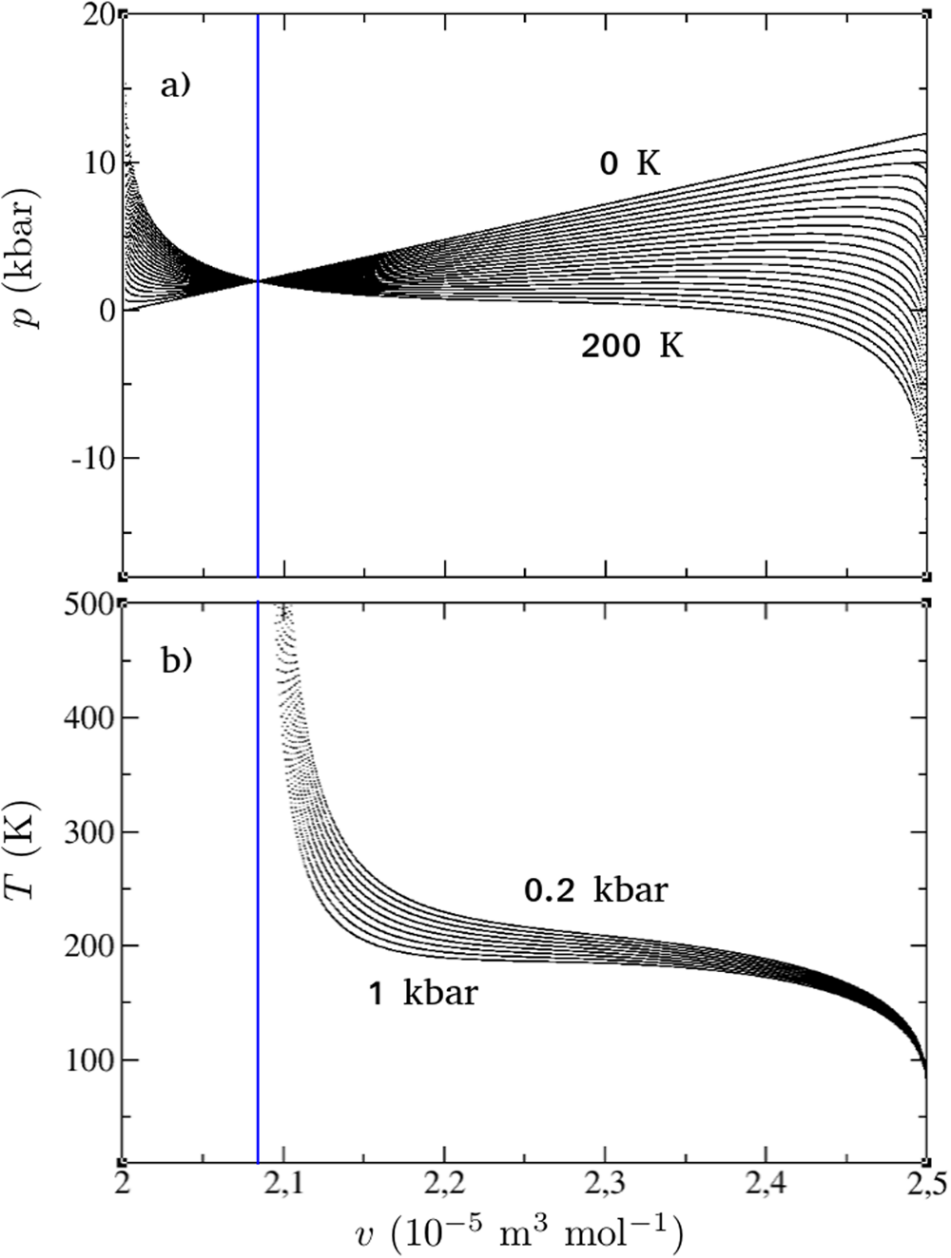
(**a**) Pressure (*p*) *versus* volume (*v*) phase diagram obtained from Eq. (3) for different values of temperature. The temperature was uniformly varied from 0 to 200 K, with steps of 10 K. The straight line is related to *T* = 0 K. Similar results were reported in ref.^12^. (**b**) Temperature (*T*) *versus* volume (*v*) for different values of pressure, which were also varied uniformly as in panel (a). The parameters used were the same as in^12^, namely $c=6$, $\delta \varepsilon =1000\,{\rm{J}}\,{{\rm{mol}}}^{-1}$, ${v}_{0}=(2\times {10}^{-5})\,{{\rm{m}}}^{3}\,{{\rm{mol}}}^{-1}$, $\delta v=(0.5\times {10}^{-5})\,{{\rm{m}}}^{3}\,{{\rm{mol}}}^{-1}$ and $\lambda =0.2$. The blue solid line indicates the lower physically valid volume in our analysis.

**Figure 3:**
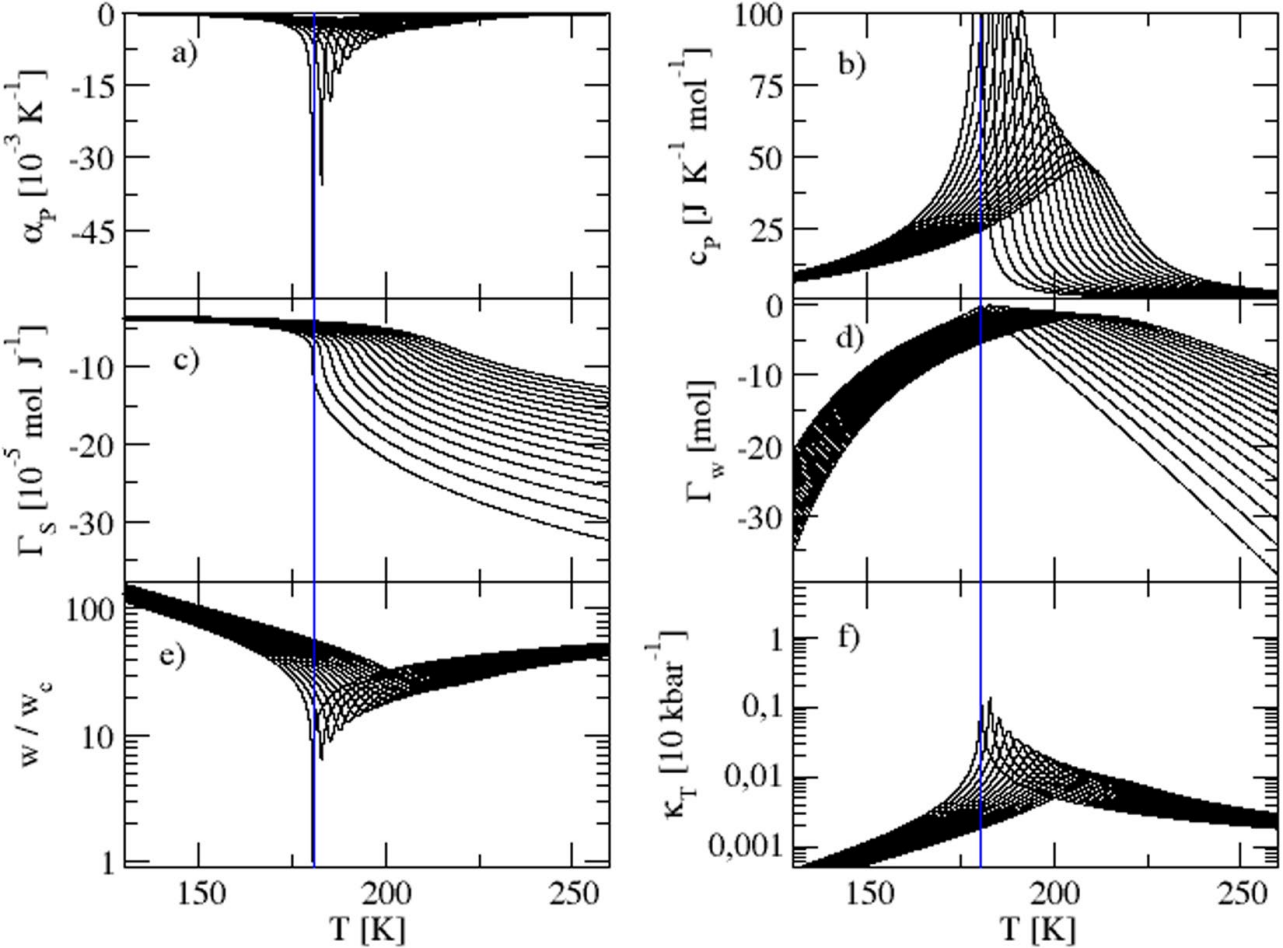
(**a**) Isobaric thermal expansivity *α*_*p*_, (**b**) isobaric heat capacity *c*_*p*_, (**c**) Grüneisen parameter ${{\rm{\Gamma }}}_{S}={\alpha }_{p}/{c}_{p}$, (**d**) Grüneisen parameter ${{\rm{\Gamma }}}_{w}={w}^{2}{\alpha }_{p}/{c}_{p}$, (**e**) speed of sound *w* normalized by its value on the critical point, namely ${w}_{c}\approx 6.513\,{\rm{m}}\,{{\rm{s}}}^{-1}$, (**f**) isothermal compressibility ${\kappa }_{T}$ for different values of pressure. The employed parameters were the same as presented in the caption of Fig. 2. The critical temperature is indicated by the vertical blue solid lines. Further details are discussed in the main text.

The obtained expressions for the observables (see Methods), namely the isobaric thermal expansion *α*_*p*_, the isobaric heat capacity *c*_*p*_ and the isothermal compressibility ${\kappa }_{T}$ together with ${{\rm{\Gamma }}}_{s}$ enable us to study the behavior of the system on the verge of the critical point. Because the equations for *c*_*p*_, *α*_*p*_, ${\kappa }_{T}$ and *T* depend on the pressure and volume of the system, they constitute a parametric system. This characteristic of the model prevents us from obtaining an analytical expression for *v*. Note that Eq. (3) (Methods) clearly indicates a transcendental equation for *v*. We thus analyze the behavior of the various observables by varying *v*, which causes variations in *T*. We fix the critical point parameters by employing the corresponding expressions, see Methods. The parameters were adjusted^12^ so that ${T}_{c}\simeq 180\,{\rm{K}}$, which is in the No Man’s Land region^2^, but a bit lower than *T*_*c*_ reported in ref.^7^ and that shown in Fig. 1. Note that the free parameters of the model reported in ref.^12^ could be changed in order to explore other systems of interest. Here, we focus on the analysis of ${{\rm{\Gamma }}}_{s}$ and ${{\rm{\Gamma }}}_{w}$ (see Methods) to the supercooled phase of water. Also, for the sake of completeness, we stress that we have recalculated both thermal expansion and specific heat, already reported in ref.^12^. Figure 2(a,b) show the *p*–*v* phase diagram for a range of temperatures and the *T*–*v* diagram. Note that when *T* = 0 K the resulting mapping $p(T=0,v)$ is a straight line. This is obtained using Eq. (3). When the temperature is high, the pressure for $v\approx {v}_{0}$ is higher than the case for low temperatures. For $v\approx {v}_{0}+\delta v$, however, higher temperatures decrease the pressure for fixed values of *v*. Figure 2(b) shows that in a particular range of values of volume, for given pressure values, physical temperature values are inaccessible. Figure 2(a) shows that the point where the pressure is the same for every temperature value (blue vertical line) is the limiting value for the volume (*v*) for which physical values of the temperature are obtained. As discussed above, we cannot analytically obtain an expression $v(T,p)$ because Eq. (3) is transcendental in *v*. Hence, we have a mapping of these physical quantities [see Eq. (4)], and we can find the corresponding *v* and *T* values for each pressure value (*p*). The same holds true for any other desired order of these three parameters. Figure 3(a–f) depict the behavior of the observables for the system considering 16 pressure values, varied in uniform steps from *p* = 1.17 kbar to 0.17 kbar. The panels a) and b) show the observables *α*_*p*_ and *c*_*p*_, which were presented and discussed in ref.^12^ for a different range of pressure values. Here, we focus on an analysis of these observables near the critical point. Remarkably, the absolute values of *α*_*p*_ and *c*_*p*_ increase significantly for $p={p}_{c}$ and $T={T}_{c}$, a fingerprint of a phase transition and/or critical point. Figure 3(c) shows the behavior of ${{\rm{\Gamma }}}_{s}={\alpha }_{p}/{c}_{p}$, see Methods. Note the effect of pressure on ${{\rm{\Gamma }}}_{s}$ and its distinct behavior upon approaching the critical point, when comparing with *c*_*p*_ and *α*_*p*_. In the immediate vicinity of the critical point, ${{\rm{\Gamma }}}_{s}$ is extremely sensitive to thermal fluctuations. Figure 3(d) shows the so-called pseudo-Grüneisen parameter ${{\rm{\Gamma }}}_{w}={w}^{2}{{\rm{\Gamma }}}_{s}$^18^ (see Methods). Note that for the data set corresponding to $p={p}_{c}$, ${{\rm{\Gamma }}}_{w}\to 0$ for $T={T}_{c}$. The vanishing of ${{\rm{\Gamma }}}_{w}$ can be understood in terms of the behavior of the normalized speed of sound *w*/*w*_*c*_ (where ${w}_{c}\approx 6.513\,{\rm{m}}\,{{\rm{s}}}^{-1}$), shown in Fig. 3(e). In the vicinity of the critical point, ${w}_{c}\to 0$, whereas its value for temperatures far from *T*_*c*_ increases to approximately 100*w*_*c*_. Physically, this finding suggests that, near the critical point, the propagation of sound waves is significantly suppressed. Interestingly, an anomalous behavior of the sound velocity was also observed close to the Mott critical end-point in strongly correlated electronic systems and associated with a diverging compressibility of the electronic degrees of freedom^19,20^. Figure 3(f) shows that the compressibility also presents an enhanced behavior near the critical point. Our findings are in perfect agreement with those reported in ref.^21^ for the various observables.

The Maxwell-relation ${(\frac{\partial v}{\partial T})}_{p}=-\,{(\frac{\partial S}{\partial p})}_{T}$ and the negative thermal expansivity shown in Fig. 3 indicate that the entropy (*S*) of the system is enhanced when approaching the liquid-liquid critical point, i.e., by applying pressure, the high- and low-density phases mix and the entropy increases. It is noteworthy to point out that we also find this in the finite-*T* critical end-point reported for molecular conductors^8,9^ and the quantum critical points in heavy-fermion compounds^22,23^. The high- and low-density phases produce two different energy scales. Because the degree of H-bonding depends on temperature and pressure, a scaling cannot be applied successfully^24,25^. Reference^6^ indicates that water molecule interactions create an open H-bond structure that has a lower density than other configurations. We can capture the energy scales associated with the H-bond configurations that correspond to the low- and high-density phases using a compressible Ising-like model and two accessible system volumes. In particular, the capture of the energy scales associated with H-bonds is, in our analysis, represented by the vanishing of one of the possible volumes associated with the sites. Using the Landau theory^26^, we find that, by decreasing the order parameter fluctuations, a divergence in both the correlation length^7^ and relaxation time^27^ are expected. Reference^28^ reports a connection between the entropy-dependent relaxation time and ${{\rm{\Gamma }}}_{s}$. We here suggest that this is also true for supercooled water.

**Figure 4:**
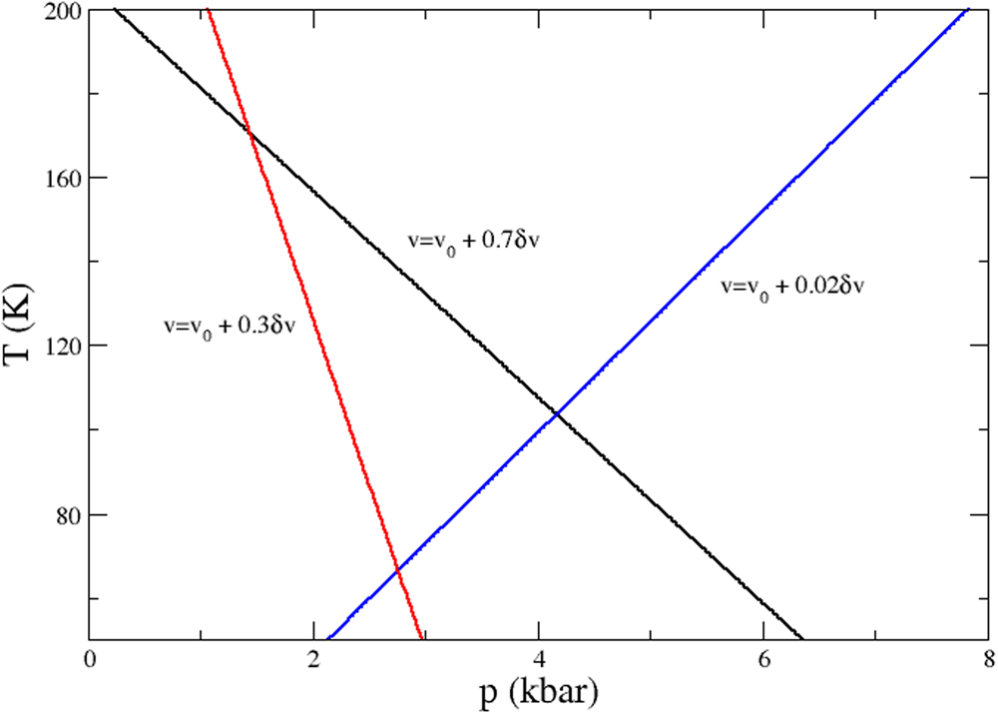
Temperature (*T*) *versus* pressure (*p*) phase diagram for three different values of volume (*v*), as indicated in the label. A linear relation is observed between *T* and *p* for all values of *v*. For values close to the upper limit of the volume, the pressure reaches negative values and the angular coefficient of the mathematical relation between *T* and *p* changes sign. For high values of *v* (more precisely, for $v > {v}_{0}+0.17\delta v$ in the case where $\lambda =0.2$), the angular coefficient is negative, indicating a decrease in temperature as pressure increases. The values of *v*_0_ and *δv* employed here are the same as in Fig. 2.

In what follows, we use the compressible cell Ising-like model to study the Ising-nematic phase recently detected in the low-doping regime of Fe-based superconductors^29^. An electronic nematic phase is essentially a melted stripe phase^30^. Figure 4 shows that as the pressure is increased for $v={v}_{0}+\delta v$, the temperature decreases. The limiting volume value for such a behavior is $v={v}_{0}+0.17\delta v$ for $\lambda =0.2$.

In the case of the proposed nematic phase in Fe-based superconductors, the pressure variation is caused by the chemical pressure introduced in the system by the doping effect on the crystal lattice. As the pressure (doping) is varied, the critical point signature vanishes (see Fig. 3). We obtain the same behavior shown in Fig. 4 (red curve) experimentally for the 122 doped Fe-based superconductors^31^. In particular, the thermal expansion signatures are suppressed upon doping^31^. Comparing the pressure *versus* temperature phase diagram reported in ref.^31^ for the 122 doped Fe-based superconductor with our results, we see that the regime that better illustrates the nematic phase is the one where $v={v}_{0}+0.7\delta v$, since the critical point signature is shifted for lower values of *T* as *p* increases (see Fig. 4). Because there is a substantial number of free parameters that compose the current Ising-like model, we leave the fitting of the experimental results reported in ref.^31^ to future research. Here we used the compressible cell Ising-like model to simulate the doping effect in single crystals by assuming there are only two different volumes in the melted electronic nematic phase^30^. When the system is doped, the electronic nematic phase associated with two coexisting volumes (see the figure in ref.^30^) is suppressed, and the reported superconductivity appears, e.g., for Ba(Fe_1−*x*_Co_*x*_)_2_As_2_ single crystals^31,32^. Yet, it is worth mentioning that for the limit case where $v={v}_{0}+0.17\delta v$, we have no pressure variations for a wide range of temperature values. Since pressure and volume are conjugated variables, this behavior can be associated to the Invar effect, which has been widely investigated in iron-nickel alloys, see e.g.^33^.

Finally, we highlight our main findings. We have used an energy-volume coupled Ising-like model to calculate the Grüneisen parameter for the liquid-liquid transition in supercooled water^12^. We find that the behavior of the Grüneisen parameter is enhanced near pressure and temperature values that display anomalous behavior and thus supports the presence of a liquid-liquid critical point governed by two distinct energy scales. Yet, such proposal is corroborated by the singular behavior of the isothermal compressibility, sound velocity and pseudo-Grüneisen parameter in the vicinity of the liquid-liquid critical point. Since the first submission of this manuscript, the compressible cell Ising-like model employed here has been used to describe the two-critical-point scenario^34^. In addition to exploring the critical behavior of water and its other phases, our model can also be applied to other systems by adjusting its parameters. The application of the model to describe the nematic phase in the low-doping regime of Fe-based superconductors revealed that the low-doping regime is well-described by choosing values near the upper boundary values of the volume of each cell, namely, $v\approx {v}_{0}+\delta v$. The latter corresponds to a lower-density configuration, in agreement with the theoretical description of the nematic phase for Fe-based superconductors^32^. Our analysis of the Grüneisen parameter ${{\rm{\Gamma }}}_{s}$ and pseudo-Grüneisen parameter ${{\rm{\Gamma }}}_{w}$ can be applied to investigate the critical behavior in any two-state system. One needs only to adjust properly the critical parameters according with the system of interest.

## Methods

We recall some of the results obtained in the model proposed in ref.^12^, which consist the basis of our analysis.

The system has *N* sites and coordination number *c*, where *c* is an adimensional parameter responsible for dictating the influence of the interaction among the sites when compared to its intrinsic energy $cN{\varepsilon }_{0}/2$, where ${\varepsilon }_{0}$ is an arbitrary energy value. In each site, we suppose the existence of a cell. Each cell is characterized by its volume (and, consequently, its density). The interaction between sites is dictated by a constant energy coupling $\delta \varepsilon$. The total energy of the system is $E\{{n}_{i}\}$, where ${n}_{i}=1$ if the volume of the respective cell is ${v}_{0}+\delta v$ and ${n}_{i}=0$ if its volume is *v*_0_. The expression for the energy reads

1 $$E\{{n}_{i}\}=\frac{cN{\varepsilon }_{0}}{2}-\delta \varepsilon \,\sum _{\langle ij\rangle }\,{n}_{i}{n}_{j}.$$

The volume of the system is the sum of the volume of each cell. Since the minimum volume that each cell occupies is *v*_0_, all cells contribute to the total volume with a magnitude of *Nv*_0_. Adding the contribution of the *K* sites having a volume ${v}_{0}+\delta v$, the expression for the total volume reads^12^

2 $$V\{{n}_{i}\}=N{v}_{0}+K\delta v,$$

Thus each particle is located in a site, and the volume has two possible values. We associate these two volumes with the low- and high-density phases and thus with two distinct energy scales. The association of different energy scales with the volume of each cell and, consequently, by their densities, is the key to understanding why ${{\rm{\Gamma }}}_{s}$ is enhanced near the liquid-liquid critical point. The energy has two boundary values, corresponding to two limiting configurations of the system. When $K=0$, ${E}_{max}=cN{\varepsilon }_{0}\mathrm{/2}$ results, whereas for $K=N$, ${E}_{min}=[cN{\varepsilon }_{0}-\delta \varepsilon (N-\mathrm{1)}N\mathrm{]/2}$. The associated minimum and maximum values for the volumes are ${V}_{min}=N{v}_{0}$ for $K=0$ and ${V}_{max}=N({v}_{0}+\delta v)$ for $K=N$. Physically, the limiting cases represent the scenarios where all cells occupy the minimum (maximum) volume, corresponding to $K=0$ ($K=N$). We obtain all the observables related to the system from Eqs (1) and (2), cf. ref.^35^. We carry out an isothermal-isobaric analysis and sum ${e}^{-E/{k}_{B}T}$ and ${e}^{-pV/{k}_{B}T}$ to the partition function, where *k*_*B*_ is the Boltzmann constant and *p* and *T* are the pressure and temperature of all possible microstates of the system, respectively.

The resulting partition function $Z=Z(N,p,T)$ has the same mathematical structure as the Ising canonical partition function. Because we have not yet solved the three-dimensional Ising model, we use an approximate *mean-field solution*^12^ to obtain the observables. The mean-field theory can be applied to a wide range of systems, including the Ising model and the van der Waals theory for liquid-gas systems^35^. Using it we replace the functional integral $Z=N\,\int \,(Dm){e}^{-E[m,H]}$ with the maximum value of the integrand, the so-called *saddle-point approximation*. The parameter *m* is the order-parameter density, and *Dm* is the volume element. Because this approximation assumes that the only important configuration near the critical point is the one of uniform density, we expect that, because the density fluctuations in the order parameter are strong in this regime, this study of critical phenomena will exhibit artifacts. However ref.^12^ indicates that consistent results can be obtained in this framework. The equation of state for the system is^12^

3 $$p(T,v)=\frac{T{k}_{B}}{\delta v}\,\mathrm{ln}\,(\lambda \frac{{v}_{0}+\delta v-v}{v-{v}_{0}})+c\frac{\delta \varepsilon }{\delta v}\frac{v-{v}_{0}}{\delta v},$$

from which we deduce

4 $$T(p,v)=\frac{\delta v}{{k}_{B}\,f(v)}[p-c\frac{\delta \varepsilon }{\delta {v}^{2}}(v-{v}_{0})].$$

We use Eq. (3) to determine the critical point coordinates ${p}_{c}=({v}_{c},{T}_{c})$, following ref.^35^:

5 $${(\frac{\partial p}{\partial v})}_{T}=0;\,{(\frac{{\partial }^{2}p}{\partial {v}^{2}})}_{T}=0.$$

Thus,

6 $${(\frac{\partial p}{\partial v})}_{T}=\frac{c\delta \varepsilon }{\delta {v}^{2}}-\frac{T{k}_{B}}{({v}_{0}+\delta v-v)\,(v-{v}_{0})},$$

and

7 $${(\frac{{\partial }^{2}p}{\partial {v}^{2}})}_{T}=T{k}_{B}[\frac{2({v}_{0}-v)+\delta v}{{({v}_{0}+\delta v-v)}^{2}{(v-{v}_{0})}^{2}}].$$

We apply these conditions and the critical point parameters are:

$${v}_{c}={v}_{0}+\frac{1}{2}\delta v;\,{T}_{c}=\frac{c\delta \varepsilon }{4{k}_{B}};\,{p}_{c}=\frac{c}{4}\frac{\delta \varepsilon }{\delta v}(2+\,\mathrm{ln}\,\lambda ).$$

Employing the basic thermodynamic relations^35^ and using $f(v)=\,\mathrm{ln}(\lambda \frac{{v}_{0}+\delta v-v}{v-{v}_{0}})$^12^ we obtain the isobaric thermal expansion *α*_*p*_, the heat capacity *c*_*p*_ and the isothermal compressibility ${\kappa }_{T}$

8 $${\alpha }_{p}=\frac{1}{v}{\{\frac{\delta {v}^{2}}{{k}_{B}f{(v)}^{2}}g(v)[p-\frac{c\delta \varepsilon }{\delta {v}^{2}}(v-{v}_{0})]-\frac{c\delta \varepsilon }{{k}_{B}\delta vf(v)}\}}^{-1},$$

9 $${c}_{p}=T\frac{{k}_{B}}{\delta v}f(v){\{\frac{\delta {v}^{2}}{{k}_{B}f{(v)}^{2}}g(v)[p-\frac{c\delta \varepsilon }{\delta {v}^{2}}(v-{v}_{0})]-\frac{c\delta \varepsilon }{{k}_{B}\delta vf(v)}\}}^{-1},$$

10 $${\kappa }_{T}=-\,\frac{1}{v}{[\frac{c\delta \varepsilon }{\delta {v}^{2}}-T{k}_{B}g(v)]}^{-1},$$

11 $$g(v)=\frac{1}{({v}_{0}+\delta v-v)\,(v-{v}_{0})}.$$

From Eqs 8 and 9 we see that the *T*-dependence of *α*_*p*_ and *c*_*p*_ are distinct. Thus, we expect a different singular behavior of these observables upon approaching the critical point, which can be explored by means of the Grüneisen parameter (see below). This is one of the main findings of this work, see Results.

We use Eqs (8) and (9) to determine the expression of the ratio ${{\rm{\Gamma }}}_{s}={\alpha }_{p}/{c}_{p}$ and the pseudo-Grüneisen parameter ${{\rm{\Gamma }}}_{w}={w}^{2}{{\rm{\Gamma }}}_{s}$^16,18,21^, where *w* is the speed of sound, with

12 $${w}^{2}=\frac{\partial p}{\partial \rho }\approx \frac{\partial p}{\partial (1/v)}.$$

The calculations are straightforward and we obtain:

13 $${{\rm{\Gamma }}}_{w}=\frac{\delta v}{T{k}_{B}v}{[\mathrm{ln}(\lambda \frac{{v}_{0}+\delta v-v}{v-{v}_{0}})]}^{-1}[\frac{T{k}_{B}{v}^{2}}{({v}_{0}+\delta v-v)(v-{v}_{0})}-c\delta \varepsilon \frac{{v}^{2}}{\delta {v}^{2}}],$$

14 $${{\rm{\Gamma }}}_{s}=\frac{\delta v}{T{k}_{B}v}{[\mathrm{ln}(\lambda \frac{{v}_{0}+\delta v-v}{v-{v}_{0}})]}^{-1}.$$

Both quantities, namely ${{\rm{\Gamma }}}_{s}$ and ${{\rm{\Gamma }}}_{w}$ were used in our analysis, see Fig. 3(c,d).
